# Advancing Minimally Invasive Mitral Valve Surgery: Early Outcomes of a Total Endoscopic 2D and 3D Approach

**DOI:** 10.3390/jcdd12120501

**Published:** 2025-12-18

**Authors:** Carlo Savini, Mariafrancesca Fiorentino, Diego Sangiorgi, Simone Calvi, Antonino Costantino, Elena Tenti, Elisa Mikus

**Affiliations:** 1 Cardiovascular Department, Maria Cecilia Hospital, GVM Care & Research, 48033 Cotignola, Italy; 2 Department of Experimental Diagnostic and Surgical Medicine (DIMEC), University of Bologna, 40126 Bologna, Italy

**Keywords:** mitral valve repair, minimally invasive cardiac surgery, total endoscopic cardiac surgery

## Abstract

Background: The minimally invasive approach is increasingly recognized as the standard for surgical management of mitral valve disease. Advances in endoscopic visualization and surgical instrumentation have enhanced precision while minimizing trauma, improving both functional and esthetic outcomes. This study presents a single-center experience with total endoscopic mitral valve repair (MVR) performed using two- or three-dimensional video-assisted technology. Methods: Between October 2022 and September 2025, 239 patients underwent total endoscopic MVR at our institution. Demographic, operative, and postoperative data were collected and analyzed. Results: Median age was 63 years, with 64.4% male. Median logistic EuroSCORE and EuroSCORE II were 2.53 and 0.83, respectively. Most patients were NYHA class II (54.4%), and 47.7% had pulmonary hypertension. Mitral annuloplasty was performed in 99.2% of cases; 78.6% received Gore-Tex chordae, 6.3% underwent posterior leaflet resection, and 11.7% edge-to-edge repair. Conversion to sternotomy occurred in 0.4%. In-hospital mortality was 1.3%; stroke occurred in 0.4%. Postoperative atrial fibrillation developed in 26.8%, while major complications such as sepsis (2.1%) and renal failure requiring dialysis (1.3%) were infrequent. Median ventilation time was 5 h, ICU stay was 2 days, and hospital stay was 7 days. Pre-discharge echocardiography showed ≤mild regurgitation in 99.2%. Conclusions: Total endoscopic MVR using two- or three-dimensional video assistance is safe, feasible, and yields excellent clinical, functional, and cosmetic results, with low morbidity and rapid recovery.

## 1. Introduction

Mitral regurgitation (MR) is the second most common valvular heart disease in Western countries [1]. According to international guidelines, mitral valve repair is the treatment of choice whenever the valve anatomy and underlying mechanism predict a durable outcome [2,3], providing superior long-term survival compared with valve replacement, whether mechanical or bioprosthetic [4]. Over the past decades, cardiac surgery has undergone a profound evolution. Traditional open-heart surgery via full median sternotomy has been progressively complemented by minimally invasive techniques designed to reduce surgical trauma without compromising safety or efficacy [5]. These approaches, characterized by smaller incisions, limited surgical exposure, and specialized instrumentation, aim to facilitate faster recovery and improved cosmetic outcomes while maintaining excellent short- and long-term results [6,7,8]. In mitral valve surgery, the right mini-thoracotomy approach has replaced median sternotomy in many centers, significantly reducing surgical trauma and becoming the standard approach for mitral valve repair. In this context, advances in surgical instrumentation and visualization represent key tools for enhancing precision and reducing biological invasiveness. The introduction of high-definition two- and three-dimensional endoscopic visualization systems has further expanded the concept of minimal invasiveness in mitral valve surgery. These technologies allow for surgical access through even smaller incisions while offering an exceptionally realistic and magnified view of the mitral valve, enabling the surgeon to accurately assess the complex anatomy of the subvalvular apparatus in its native geometric relationships. This enhanced visual fidelity facilitates precise suturing and leaflet manipulation, improving technical accuracy and potentially reducing operative errors.

This study aims to present our single-center experience with totally endoscopic mitral valve repair using two- and three-dimensional video-assisted technology, focusing primarily on early clinical outcomes, including operative mortality. Secondary objectives include evaluating procedural feasibility, safety, and perioperative results, providing a comprehensive overview of the initial experience with this minimally invasive approach.

## 2. Materials and Methods

### 2.1. Study Population

All adult patients (≥18 years) who underwent totally endoscopic mitral valve repair using two- or three-dimensional video-assisted technology at our institution between October 2022 and September 2025 were retrospectively included in the present analysis. No formal sample size calculation was performed; instead, all consecutive eligible cases during the study period were considered. The selection of a fully endoscopic approach was carefully individualized and made at the discretion of the operating surgeon, based on detailed preoperative AngioCT evaluation. Key factors considered included the presence of aortic calcifications or soft plaques and the diameter of the femoral vessels, which could influence procedural safety and feasibility. Additionally, patients with prior surgery on either lung were excluded from the minimally invasive approach, as single-lung ventilation is required and may not be safely tolerated in these cases. During the same period, a total of 697 mitral valve repairs were performed at our center, of which 472 were carried out using a minimally invasive technique. Among these, 239 patients underwent a fully endoscopic procedure and constituted the study population. The study was conducted in compliance with the principles of the Declaration of Helsinki. Ethical approval was obtained from the Romagna Ethics Committee on 20 November 2019 (Prot. 9689/2019 I.5/186). Owing to the retrospective design, the requirement for individual informed consent was waived. Preoperative, intraoperative, and postoperative clinical data were retrieved from institutional medical records, with rigorous efforts made to minimize missing information; only complete datasets were included in the final analysis. The primary endpoints were in-hospital mortality—defined as any death occurring before discharge from the index hospitalization—, the incidence of postoperative adverse events, and the need for conversion to full sternotomy. Furthermore, a dedicated risk factor analysis was conducted for the most frequent postoperative complications, specifically the occurrence of new-onset atrial fibrillation and the requirement for red blood cell transfusion.

### 2.2. Operative Strategy

**Figure 1:**
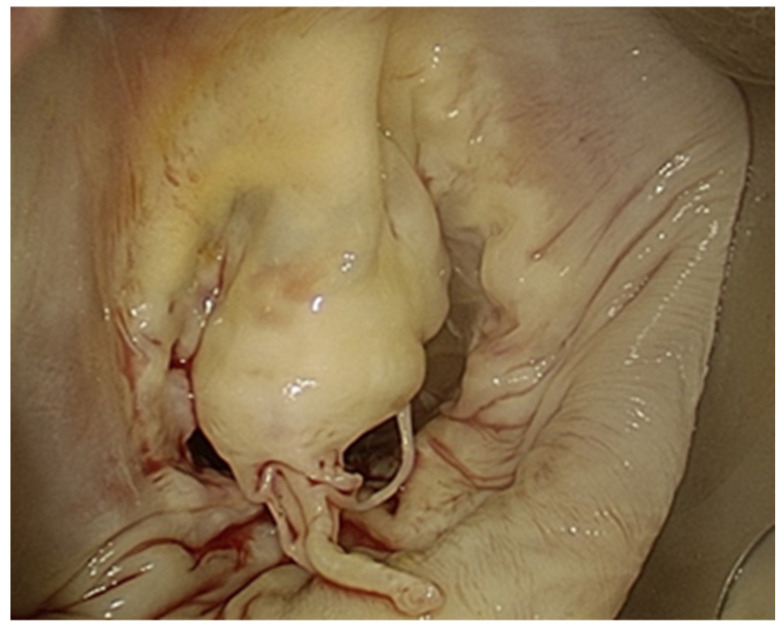
Intra-operative view of mitral valve flail.

Surgical indications were determined according to the latest guidelines of the European Society of Cardiology and the European Association for Cardio-Thoracic Surgery [2,3]. The decision to perform a totally endoscopic procedure was made at the discretion of the operating surgeon, guided by preoperative thoraco-abdominal AngioCT imaging. All patients underwent CT assessment to identify vascular calcifications or wall abnormalities that could compromise the safety of peripheral femoral cannulation. When femoral access was deemed unsafe but an endoscopic approach was still considered appropriate, arterial cannulation was instead performed through the axillary artery. Our institutional technique for minimally invasive mitral valve repair through right mini-thoracotomy has been previously detailed [9,10]; however, several technical aspects differ for the totally endoscopic approach. All procedures were conducted under total intravenous anesthesia with selective single-lung ventilation achieved via a double-lumen endotracheal tube. Transesophageal echocardiography (TEE) was routinely employed for preoperative assessment and continuous intraoperative monitoring of cardiac and valvular function. Peripheral cardiopulmonary bypass (CPB) was established through a 3 cm groin incision. The femoral artery and vein were cannulated using the Seldinger technique under TEE guidance. In cases requiring axillary access, a small deltopectoral incision was made to expose and directly cannulate the axillary artery through a surgical arteriotomy, while venous drainage was obtained percutaneously via the femoral vein. In female patients, a small incision was placed along the inframammary crease; in male patients, a similar incision was created either at the level of the fourth right intercostal space or in a peri-areolar position. A right anterior mini-thoracotomy was then performed through the fourth intercostal space. Two accessory ports were placed: one in the sixth intercostal space for intracardiac suction and CO_2_ insufflation, and another in the same intercostal plane as the thoracotomy for endoscopic visualization. CPB was initiated, the pericardium was opened approximately 4–5 cm anterior to the phrenic nerve, overlying the right ventricle, and retracted laterally using traction sutures passed externally with an EndoClose device, allowing optimal exposure beneath the camera line. A Cooley needle for aortic venting and cardioplegia administration was positioned in the ascending aorta, and cross-clamping was achieved exclusively with a Chitwood clamp introduced through a 10 mm incision in the second intercostal space and advanced into the transverse sinus. Myocardial protection was provided using Custodiol cardioplegia delivered antegradely into the aortic root. Custodiol^®^ cardioplegia was administered as a single-dose infusion at the onset of aortic cross-clamping, providing myocardial protection for up to 180 min; a repeat dose was only given if the cross-clamp time exceeded this interval. When tricuspid valve surgery was required, caval snares were applied around the venous cannulae before right atriotomy to optimize visualization. After achieving cardioplegic arrest, the left atrium was opened, and the mitral valve was carefully examined to identify the mechanism of mitral regurgitation and to confirm the findings previously obtained from the transesophageal echocardiogram (Figure 1).

**Figure 2:**
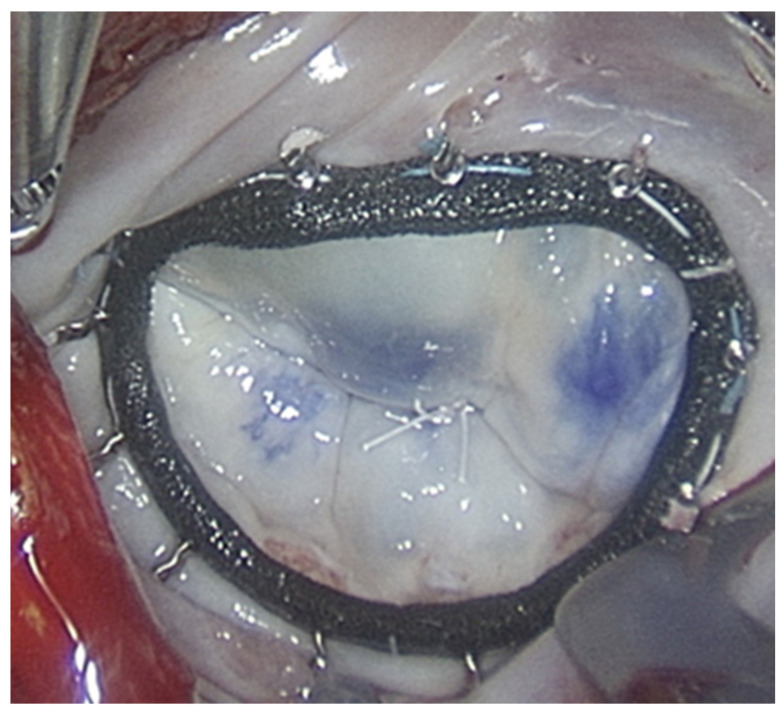
Mitral valve repair using a complete annuloplasty ring and implanting two pairs of Gore-Tex artificial chordae tendineae.

Subsequently, mitral valve repair was performed, most commonly using a complete annuloplasty ring, and implanting 4-0 Gore-Tex artificial chordae tendineae as shown in Figure 2.

If indicated, tricuspid valve procedures were carried out after left atrial closure. At the conclusion of intracardiac repair, temporary epicardial pacing wires were positioned on the right ventricle, the aortic clamp was released, and patients were weaned from CPB following standard institutional protocols. Postoperative management was identical to that used after conventional sternotomy procedures. Upon admission to the intensive care unit (ICU), patients were evaluated for early extubation on postoperative day (POD) 0, depending on chest tube output, neurological status, and gas exchange. If clinically stable, patients typically remained in the ICU overnight and were transferred to a medium care unit (MCU) on POD 1. Early mobilization to a chair was encouraged on POD 1, chest drains were generally removed on POD 2, and patients were then transferred to the general ward for full ambulation. Hospital discharge was typically achieved around POD 7 in the absence of postoperative complications.

### 2.3. Statistical Analysis

Continuous variables were presented as medians with interquartile ranges (IQR), and categorical variables were expressed as absolute numbers and frequencies. Multivariable logistic regression was conducted to assess postoperative atrial fibrillation or transfusion, using baseline and intraoperative characteristics as covariates. For variable selection, the Least Absolute Shrinkage and Selection Operator (LASSO) method with 10-fold cross-validation was employed; in order to avoid possible overfitting, we selected the lambda with 1 Standard Error rule to select the most parsimonious model. The model’s discrimination was evaluated by reporting the Area Under the Receiver Operating Characteristic (ROC) Curve, while model calibration was assessed using the Hosmer–Lemeshow test. Missing data were imputed using random forest imputation for categorical variables and predictive mean matching for continuous variables. The imputation was performed with 100 trees and 5 iterations, using predictive mean matching with k = 3 to ensure realistic values for continuous variables; missing data rates ranged across variables from 0 to 2.5%. All analyses were performed using R version 4.5.0 (R Foundation for Statistical Computing, Vienna, Austria), with *p*-values < 0.05 considered as statistically significant.

## 3. Results

### 3.1. Patients’ Characteristics

Baseline patient characteristics are summarized in Table 1. The median (IQR) age was 63 (55–69) years, and 154 patients (64.4%) were male. Mitral valve disease was of degenerative origin in 199 patients (83.3%); in addition, Barlow disease was present in 29 patients (12.1%), functional atrial in 4 patients (1.7%), endocarditis in 6 patients (2.5%), and rheumatic disease in 1 patient (0.4%). Among the degenerative cases, 54 patients (22.6%) had bileaflet prolapse, 8 (3.3%) an isolated anterior prolapse, 36 (15.1%) an isolated posterior prolapse, and 83 (34.7%) an isolated P2 flail. The remaining patients presented flail involving multiple segments of the posterior leaflet. In our cohort, severe tricuspid regurgitation (TR) was present in 7 patients (2.9%), moderate TR in 43 patients (18%), mild TR in 167 patients (69.9%), and no tricuspid regurgitation in 22 patients (9.2%). The most prevalent cardiovascular risk factors were hypertension (46.9%) and dyslipidemia (42.7%). At the time of surgery, the majority of patients were in New York Heart Association (NYHA) functional class II (54.4%) or I (36%), while seven patients (2.9%) presented with acute heart failure. Chronic obstructive pulmonary disease (COPD) was observed in 10 patients (4.2%), and a previous history of stroke or myocardial infarction was recorded in two patients (0.8%). Renal function was generally preserved, with a median (IQR) serum creatinine level of 0.89 (0.78–1.00) mg/dL. The median (IQR) preoperative EuroSCORE II was 0.83 (0.61–1.25). Preoperative atrial fibrillation was documented in 43 patients (17.6%), and pulmonary hypertension (systolic pulmonary artery pressure > 30 mmHg) was present in 114 patients (47.7%).

### 3.2. Intraoperative Characteristics

Intraoperative characteristics are reported in Table 2. Almost all patients (99.2%) underwent mitral valve annuloplasty, with 78.6% receiving Gore-Tex chordae implantation, 6.3% undergoing posterior leaflet resection, and 11.7% treated with edge-to-edge suturing. Concomitant procedures were relatively uncommon (16.3%), including PFO closure in 25 patients (10.5%), left atrial appendage exclusion in 12 patients (5%), and concomitant tricuspid valve repair in 11 patients (4.6%). Median cardiopulmonary bypass (CPB) and aortic cross-clamp times were 175 min (IQR 155–195.5) and 114 min (IQR 100–133), respectively. Only one patient (0.4%) required conversion to full sternotomy due to iatrogenic aortic dissection, and similarly, only one patient out of 239 (0.4%) required a second pump run. There were no mitral repair failures or intraoperative deaths. Thirteen patients (5.4%) required inotropic support due to difficulty weaning from cardiopulmonary bypass, and 4 patients (1.7%) underwent intra-aortic balloon pump implantation.

### 3.3. In-Hospital Outcomes

In-hospital outcomes are summarized in Table 3. The overall in-hospital mortality was 1.3%, with one patient (0.4%) experiencing a stroke. Two patients (0.8%) required tracheostomy due to respiratory failure, while five patients (2.1%) needed prolonged ventilation or reintubation due to pneumonia, and five patients (2.1%) developed sepsis. Perioperative myocardial infarction, defined as a new Q-wave in the electrocardiogram, creatine kinase-MB (CPK-MB) ≥ 50 UI/L or a new wall motion abnormality on echocardiography, occurred in six patients (2.5%), and three patients (1.3%) required dialysis for renal failure. New onset postoperative atrial fibrillation was observed in 64 patients (26.8%), and two patients (0.8%) required pacemaker implantation for third-degree AV block. Re-thoracotomy for bleeding was necessary in nine patients (3.8%), and 39.3% of patients received packed red blood cell transfusions. Median ventilation time was 5 h, median ICU stay was 2 days, and median postoperative length of stay was 7 days. Pre-discharge echocardiography showed trivial or mild mitral regurgitation in 99.2% of patients.

Starting from July 2023, all procedures were performed using the 3D endoscopic visualization system, which progressively replaced the previous 2D technology. A descriptive overview of operative times and early outcomes between the two subgroups is provided for completeness (Table 4). In detail, we found that procedures performed with the 3D system were associated with significantly shorter aortic cross-clamp times and lower postoperative troponin levels, the latter likely reflecting the reduction in ischemic time.

### 3.4. Risk Factor Analysis

**Figure 3:**
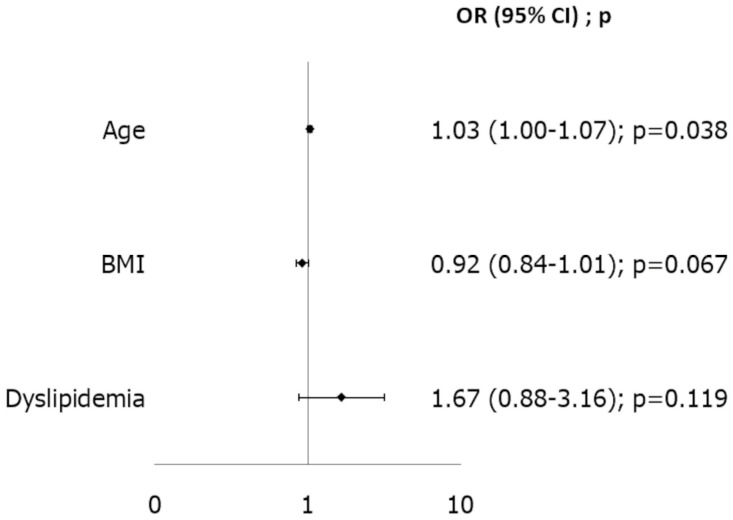
Risk factor analysis for postoperative Atrial fibrillation. BMI: Body Mass Index.

**Figure 4:**
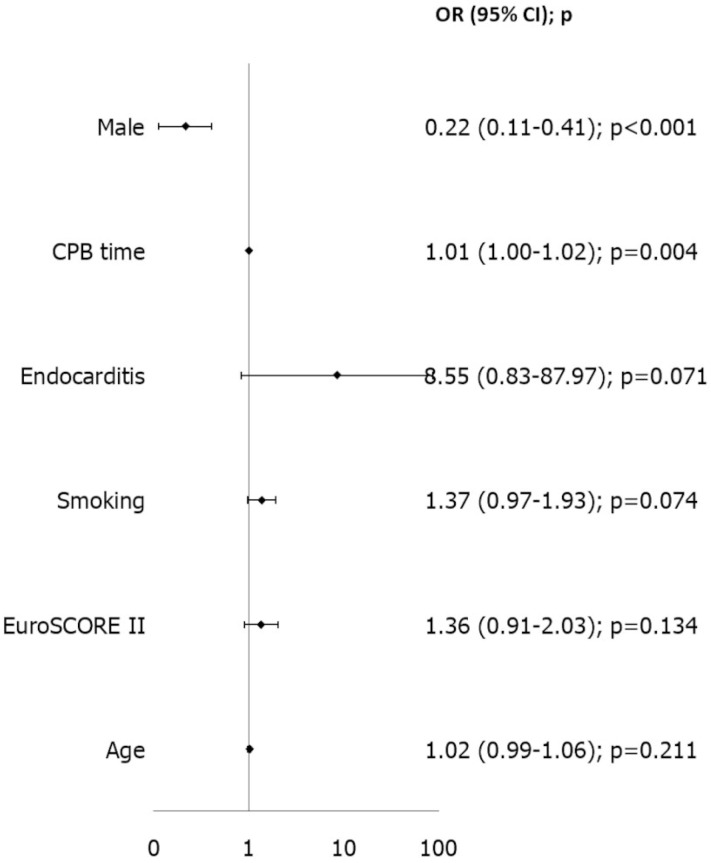
Risk factor analysis for postoperative Transfusions. CPB: CardioPulmonary Bypass.

Subsequently, we performed an analysis of risk factors for the most frequent adverse events in our population. Due to the low incidence of mortality and major complications such as stroke, renal failure, and sepsis, we focused on identifying risk factors for postoperative atrial fibrillation (AF) and for the need for packed red blood cell transfusions. As described in the statistical analysis section, variable selection was performed using the Least Absolute Shrinkage and Selection Operator (LASSO) method with 10-fold cross-validation. Multivariable analysis identified age as the only independent risk factor for postoperative AF [OR 1.033 (95% CI 1.002–1.065), *p* = 0.038]. The discriminatory ability of this model was modest, with an area under the curve (AUC) of 0.649; the Hosmer–Lemeshow test *p*-value was 0.125 (Figure 3). Independent risk factors for the need for postoperative transfusions were cardiopulmonary bypass time [OR 1.013 (95% CI 1.004–1.021), *p* = 0.004], whereas male sex was found to be protective against the need for transfusions [OR 0.216 (95% CI 0.114–0.407), *p* < 0.001]. The discriminative performance of the transfusion model was better, with an AUC of 0.773 and a Hosmer–Lemeshow test *p*-value of 0.272 (Figure 4).

## 4. Discussion

In this study, we report our experience with totally endoscopic mitral valve repair, representing the natural evolution of minimally invasive surgery. By avoiding median sternotomy and utilizing a small right anterolateral thoracic incision, this approach combines reduced surgical trauma with enhanced and magnified visualization of the mitral valve. This aligns with the broader trend toward less invasive cardiac procedures without compromising repair safety or durability. The 2025 guidelines on valvular heart disease [3] acknowledge that minimally invasive approaches may be considered in experienced, high-volume centers, supported by evidence demonstrating comparable outcomes to sternotomy-based repair [5,6] and benefits such as shorter ventilation, ICU and hospital stays, reduced postoperative pain, and improved patient comfort [7]. Within minimally invasive mitral surgery, several approaches coexist. While some centers rely on direct vision, others employ totally endoscopic techniques. Although no single method is universally superior, the endoscopic approach provides realistic, magnified visualization of leaflet and subvalvular anatomy, improved suturing precision, and a naturally educational environment, as each procedural step is displayed on-screen. Three-dimensional optical systems further enhance depth perception and spatial orientation, potentially improving accuracy and ergonomics. The thoracoscopic approach has been widely reported in high-volume centers. Jiang et al. [11] and Cheng et al. [12] demonstrated the feasibility, safety, and favorable recovery profile of totally endoscopic mitral surgery. Comparative studies with sternotomy for mitral valve replacement [13] reported similar safety despite longer cross-clamp and bypass times, while consistently noting shorter ICU/hospital stays, reduced ventilation times, and decreased drainage. Similar findings were reported for mitral valve repair [14,15,16], with minimally invasive approaches associated with lower transfusion requirements, faster recovery, and higher patient satisfaction with cosmetic outcomes. Our findings are consistent: despite no direct sternotomy comparison, we observed low mortality (1.3%) and a very low incidence of major complications, while recovery—including ventilation time and ICU/hospital length of stay—was rapid, even in selected cases of active endocarditis.

Our study adds several elements to the existing literature: (1) a large, contemporary cohort of 239 patients undergoing exclusively totally endoscopic repair within a short, homogeneous timeframe by a dedicated team; (2) detailed perioperative outcome analysis confirming safety, feasibility, and reproducibility; (3) risk-factor analysis for postoperative atrial fibrillation (AF) and transfusion, providing clinically relevant predictors for patient selection and perioperative management; (4) a comparison of 2D versus 3D endoscopic visualization, an area with limited data, suggesting 3D advantages in ergonomics and technical precision; (5) inclusion of selected cases of active endocarditis, demonstrating applicability beyond degenerative disease; and (6) updated contextualization within the 2025 guidelines, clarifying the role of totally endoscopic techniques in contemporary minimally invasive mitral surgery.

Focusing on adverse events, the low incidence of mortality and major complications (stroke, renal failure, sepsis) led us to evaluate predictors of postoperative AF and transfusion. AF remains a common complication after mitral surgery, linked to increased morbidity and mortality [17]. A recent large study reported a 16.8% incidence [18]; in our cohort, AF occurred in 26.8%. While prior studies highlighted emergent procedures, mitral valve replacement, and prolonged cross-clamp as contributors, patient age was the sole independent predictor in our analysis, consistent with reports identifying age-related atrial remodeling and fibrosis as drivers of postoperative arrhythmogenesis [19]. Although the model had moderate discrimination (AUC 0.62), this finding supports targeted prophylactic strategies in older patients.

Regarding transfusion requirements, prolonged cardiopulmonary bypass, postoperative inotropic support, smoking history, and female sex were independent predictors. These results have direct clinical relevance: prolonged CPB increases hemodilution and intraoperative blood loss [20], inotropic support reflects perioperative hemodynamic instability affecting coagulation and perfusion [21], and smoking-related endothelial dysfunction further raises bleeding risk. The model performed satisfactorily (AUC 0.78), and male sex appeared protective, likely due to higher baseline hemoglobin levels [22]. Notably, overall transfusion rates remained low (39.3%), consistent with previous reports showing reduced bleeding and transfusion needs in minimally invasive approaches [23,24]. This underscores one of the intrinsic advantages of the endoscopic technique: reduced surgical trauma combined with precise visualization, facilitating meticulous hemostasis.

Our findings align with the Mini-Mitral International Registry (MMIR), which analyzed 7513 patients across 17 high-volume centers in “Endoscopic and direct vision approaches in minimally invasive mitral and tricuspid valve surgery” [25]. The registry found that while totally endoscopic and robotic procedures have longer cross-clamp and bypass times, perioperative mortality and stroke were comparable to direct vision. Technical success exceeded 96%, with low conversion rates and minimal major complications [26]. In our experience, 3D endoscopy offered tangible benefits: improved depth perception enhanced leaflet manipulation, ergonomics, and handling of complex repairs. Together with MMIR data, this suggests 3D endoscopy may optimize visualization, precision, and clinical recovery, especially in high-volume, experienced centers.

These findings highlight the clinical relevance of the totally endoscopic approach and the importance of appropriate patient selection, structured team expertise, and tailored perioperative strategies. Success is influenced by the learning curve, with proficiency in minimally invasive mitral surgery estimated at approximately 75–100 cases based on CUSUM analyses of operative, bypass, and cross-clamp times, and technical failures [27,28]. In our cohort, procedural efficiency and repair complexity improved as the team became familiar with 3D optics and single-shaft instrumentation. The low conversion rate supports reproducibility once the initial learning phase is overcome. Multicenter studies with standardized reporting of surgeon experience would help refine learning-curve models and identify modifiable factors for broader adoption.

Finally, robotic-assisted versus totally endoscopic MVR remains a relevant consideration. Robotic platforms provide enhanced dexterity and ergonomics but incur higher costs and require dedicated infrastructure [29,30]. The totally endoscopic approach, using conventional instruments, may be more accessible in centers without robotic systems. Selection should consider valve complexity, resources, and surgeon experience. Our data demonstrate that excellent outcomes are achievable without robotic assistance, provided a structured program and advanced imaging, including 3D endoscopy, are in place.

Future directions include:

- development of multicenter registries to validate predictors of AF and transfusion in heterogeneous populations;
- standardized reporting of surgeon experience to refine learning-curve models;
- prospective randomized or cluster-randomized comparisons of robotic-assisted versus totally endoscopic MVR;
- dedicated evaluation of 3D versus 2D endoscopy regarding precision, operative efficiency, and clinical outcomes.

### Limitations

This study has several limitations that should be acknowledged. First, its retrospective and observational design inherently carries potential selection and information biases. Moreover, the data reflect the experience of a single institution, where—once patients were deemed suitable for a right minithoracotomy—the decision to perform a totally endoscopic or direct-visual minimally invasive approach was primarily influenced by the individual surgeon’s preference and expertise, which may limit the generalizability of the findings. Another limitation is the absence of a direct comparison with patients undergoing mitral valve surgery via conventional median sternotomy, which prevents a definitive assessment of the relative advantages of the endoscopic approach.

The present analysis also lacks a comparison with robotic-assisted mitral valve repair, which currently represents the reference standard for fully endoscopic techniques. The relatively short follow-up period precluded assessment of mid- or long-term echocardiographic outcomes, valve durability, and survival. Additionally, functional recovery and patient-reported outcomes—such as NYHA class improvement, postoperative pain, patient satisfaction, and return-to-work interval—were not systematically collected and therefore could not be evaluated.

Future prospective, multicenter studies with extended follow-up, standardized patient selection criteria, and comprehensive functional assessment will be essential to validate these findings and further define the role of totally endoscopic mitral valve repair in contemporary cardiac surgery.

## 5. Conclusions

In our single-center experience, the totally endoscopic approach proved to be a safe and feasible technique for mitral valve repair, ensuring rapid recovery and a low rate of complications. The endoscope provides an enhanced and magnified view of the operative field, allowing precise assessment of the mitral apparatus and improving technical accuracy. The adoption of three-dimensional optical systems has further refined this approach by restoring depth perception and enhancing surgical precision. The effectiveness of minimally invasive mitral surgery depends heavily on appropriate patient selection. Detailed preoperative assessment, encompassing vascular imaging and comprehensive risk evaluation, is crucial to customize the surgical strategy and reduce perioperative complications. Larger, multicenter studies with extended follow-up are necessary to confirm these findings and further clarify the place of totally endoscopic mitral valve repair in modern cardiac surgery.

## Figures and Tables

**Table 1:** Pre-operative characteristics.

Pre-Operative Characteristics	
n	239
Sex, male, n (%)	154 (64.4)
Age, median (Q1–Q3)	63 (55–69)
BMI, median (Q1–Q3)	24.2 (21.9–26.2)
Hypertension, n (%)	112 (46.9)
Diabetes, n (%)	9 (3.8)
Dyslipidemia, n (%)	102 (42.7)
Smoking, n (%)	96 (40.1)
Chronic lung disease, n (%)	10 (4.2)
Preoperative Atrial Fibrillation, n (%)	43 (17.6)
Previous Pacemaker, n (%)	1 (0.4)
NYHA class n (%) (n, %)	
- I	86 (36)
- II	130 (54.4)
- III	22 (9.2)
- IV	1 (0.4)
Acute heart failure n (%) (n, %)	7 (2.9)
Previous stroke n (%) (n, %)	2 (0.8)
Previous myocardial infarction n (%)	2 (0.8)
Creatinine, mg/dL, median (Q1–Q3)	0.89 (0.78–1.00)
Renal Impairment (GFR < 40 mL/min) n (%) (n, %)	39 (16.3)
Previous cardiac surgery, n (%)	0 (0.0)
Infective endocarditis, n (%)	6 (2.5)
Systolic pulmonary artery pressure, median (Q1–Q3)	30 (25–37)
Pulmonary hypertension (PAPs > 30 mmHg)	114 (47.7)
EuroSCORE logistic, median (Q1–Q3)	2.53 (1.72–4.90)
EuroSCORE II, median (Q1–Q3)	0.83 (0.61–1.25)

BMI: Body Mass Index; GFR: Glomerular Filtration Rate; NYHA: New York Heart Association; PAPs: Pulmonary Artery Pressure systolic.

**Table 2:** Intra-operative characteristics.

Intra-Operative Characteristics	
n	239
Annuloplasty, n (%)	237 (99.2)
Ring, n (%)	230 (96.2)
Band, n (%)	2 (0.8)
Pericardium, n (%)	5 (2.1)
Gore-Tex chordae implantation, n (%)	188 (78.6)
Posterior leaflet resection, n (%)	15 (6.3)
Edge-to-edge suturing, n (%)	28 (11.7)
Concomitant procedures, n (%)	39 (16.3)
PFO closure, n (%)	25 (10.5)
Left atrial appendage exclusion, n (%)	12 (5)
Tricuspid valve repair, n (%)	11 (4.6)
Mitral repair failures, n (%)	0 (0)
Cross-Clamp time, median (Q1–Q3)	114 (100–133)
CPB time, median (Q1–Q3)	175 (155–196)

CPB: CardioPulmonary Bypass; PFO: Patent Foramen ovale.

**Table 3:** In hospital outcomes.

In Hospital Outcomes	
n	239
Postoperative inotropic support, n (%)	13 (5.4)
IAPB support, n (%)	4 (1.7)
Postoperative troponin peak, median (Q1–Q3)	897 (648–1380)
Post-operative Atrial Fibrillation, n (%)	64 (26.8)
Pacemaker implantation, n (%)	2 (0.8)
Stroke, n (%)	1 (0.4)
Perioperative myocardial infarction, n (%)	6 (2.5)
Re-exploration for bleeding, n (%)	9 (3.8)
Sepsis, n (%)	5 (2.1)
Multi Organ Failure, n (%)	3 (1.3)
Postoperative creatinine peak, median (Q1–Q3)	0.92 (0.79–1.15)
Dialysis, n (%)	3 (1.3)
Ventilation time, median (Q1–Q3)	5 (4–7.5)
Intubation > 24 h or reintubation, n (%)	5 (2.1)
Tracheostomy, n (%)	2 (0.8)
Re-exploration for bleeding, n (%)	9 (3.8)
Patients requiring RBC transfusions, n (%)	94 (39.3)
In-hospital mortality, n (%)	3 (1.3)
ICU days, median (Q1–Q3)	2 (2–2)
LOS days, median (Q1–Q3)	7 (7–8)

IAPB: Intra-Aortic Balloon Pump; ICU: Intensive Care Unit; LOS: Length of Stay; RBC: Red Blood Cells.

**Table 4:** Intra-operative characteristics and in-hospital outcomes for 2D versus 3D.

	2D	3D	*p*
n	62	177	
Cross-Clamp time median (Q1–Q3)	122 (106, 141)	113 (99, 131)	0.018
CPB time, median (Q1–Q3)	180 (157, 196)	174 (153, 195)	0.350
Postoperative inotropic support, n (%)	4 (6.5)	9 (5.1)	0.746
IAPB support, n (%)	2 (3.2)	2 (1.1)	0.277
Postoperative troponin peak, median (Q1–Q3)	1030 (760, 1518)	875 (619, 1371)	0.024
Post-operative Atrial Fibrillation, n (%)	16 (25.8)	48 (27.1)	1.000
Pacemaker implantation, n (%)	0 (0.0)	2 (1.1)	1.000
Stroke, n (%)	1 (1.6)	0 (0.0)	0.259
Perioperative myocardial infarction, n (%)	3 (4.8)	3 (1.7)	0.240
Re-exploration for bleeding, n (%)	4 (6.5)	5 (2.8)	0.243
Sepsis, n (%)	2 (3.2)	3 (1.7)	0.607
Multi Organ Failure, n (%)	1 (1.6)	2 (1.1)	1.000
Postoperative creatinine peak, median (Q1–Q3)	0.90 (0.76, 1.05)	0.93 (0.80, 1.17)	0.103
Dialysis, n (%)	0 (0.0)	3 (1.7)	0.570
Ventilation time, median (Q1–Q3)	5 (4, 6)	5 (4, 8)	0.410
Intubation > 24 h or reintubation, n (%)	1 (1.6)	3 (1.7)	1.000
Tracheostomy, n (%)	0 (0.0)	2 (1.1)	1.000
Re-exploration for bleeding, n (%)	4 (6.5)	5 (2.8)	0.243
Patients requiring RBC transfusions, n (%)	20 (32.3)	74 (41.8)	0.227
In-hospital mortality, n (%)	1 (1.6)	2 (1.1)	1.000
ICU days, median (Q1–Q3)	2 (2, 2)	2 (2, 3)	0.001
LOS days, median (Q1–Q3)	7 (7, 8)	7 (7, 8)	0.215

IAPB: Intra-Aortic Balloon Pump; ICU: Intensive Care Unit; LOS: Length of Stay; RBC: Red Blood Cells.

## Data Availability

The data presented in this study are available on request from the corresponding author. The data are not publicly available due to Data Protection Directive 95/46/EC.
